# Plastic Surgery in Poland—Should We be Concerned?

**DOI:** 10.1055/a-2624-1691

**Published:** 2025-07-23

**Authors:** Tomasz Korzeniowski, Jerzy Strużyna

**Affiliations:** 1 East Centre of Burns Treatment and Reconstructive Surgery in Leczna, District Hospital in Leczna, Poland; 2 Department of Plastic, Reconstructive and Burn Surgery, Medical University of Lublin, Lublin, Poland

Plastic surgery is a medical specialty that deals with repairing and reconstructing physical defects, as well as improving appearance. 1 A trend has been observed in which plastic surgery has been mainly reduced to performing aesthetic surgery in private practices. The aim of the study was to evaluate plastic surgery in Poland in terms of the department's activity, employment of physicians, and scope of procedures performed. Fifteen plastic surgery departments in Poland participated in the survey.

**Fig. 1:**
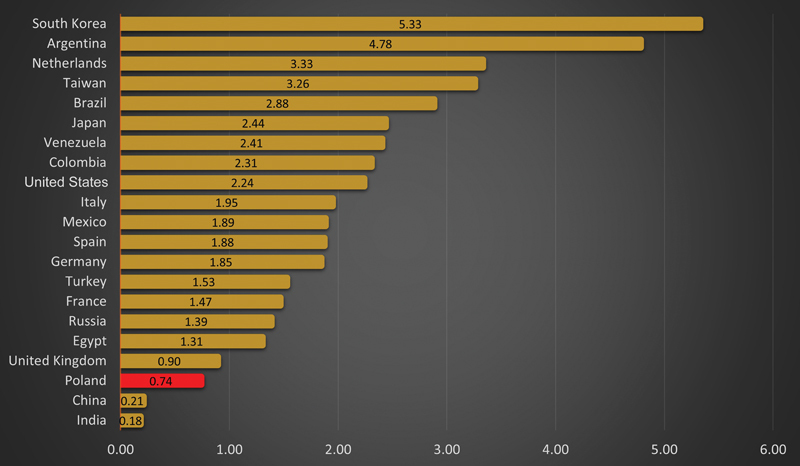
Number of plastic surgeons per inhabitant in different countries.

**Fig. 2:**
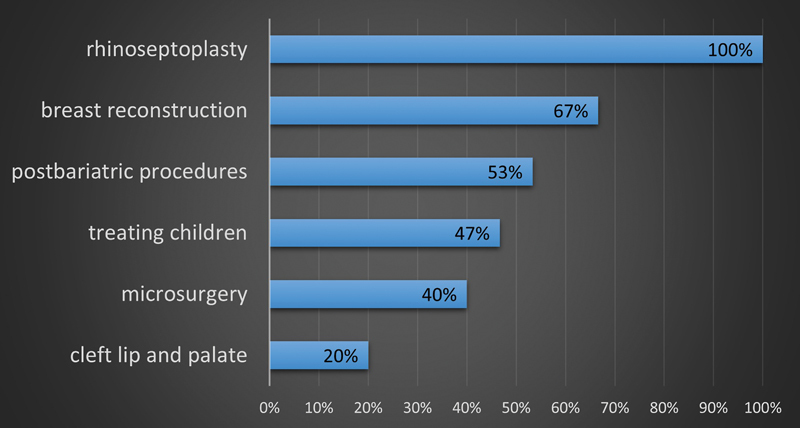
Activity of plastic surgery departments in elective cases.

A total of 282 plastic surgeons actively practice their profession, making it the least numerous surgical specialty in Poland (data from 2023). This gives the number of 0.74 specialists per 100,000 inhabitants. This ratio is much higher in most countries ( Fig. 1 ). Only 77 out of 282 (27%) plastic surgeons are employed at least in part in public hospitals. About 28 out of 282 (10%) work full-time in public hospital. Out of 15 plastic surgery departments in Poland, 8 (53%) provide 24-hour emergency service. Among these departments, all treat facial injuries, 6/8 deal with hand injuries, and 4/8 perform replantation. Only five departments treat burns. The scope of activity in elective surgery is presented in Fig. 2 .

Plastic surgery is considered a prestigious surgical specialty. Due to the very high requirements in the residency recruitment process, few succeed in becoming a plastic surgeon. For young medical students, the temptation is mainly the possibility of large salaries after completing their training, in the private sector. They mostly choose to work in private clinics, where salaries are incomparably higher than what a public hospital can offer. As U.S. research shows, higher wages in the private sector are the norm, not only in Poland. 2

A disturbing trend has been observed in the aspect of plastic surgery procedures being taken over by other surgical specialties. The results of this work confirm that most plastic surgery departments in Poland do not have a full range of activity. Many facial procedures have been taken over by maxillofacial surgeons. Cleft palate is performed by only 20% of plastic surgery departments. Treatment of defects in children is done in pediatric surgery departments. Orthopedists deal with hand surgery, ophthalmologists operate on eyelids, and otorhinolaryngologists operate on noses and ears. According to the analysis, only around one-third of the centers in Poland use microsurgical techniques. Oncologic surgeons reconstruct tumor defects in both the breast and other areas of the body.

The problem of retaining plastic surgeons in public hospitals is not only known in Poland. The American Society of Plastic Surgeons studied the size of the plastic surgery workforce to make recommendations about future needs. A survey showed that the majority of plastic surgeons in the United States (59%) choose to work in a solo practice setting. For 50% of the respondents, three-fourths or greater of their individual practice focuses on aesthetic surgery. 3 Another U.S. study found that nearly half of residents chose a career path in private practice. 4

Given these considerations, one may wonder if plastic surgery beyond the aesthetic part is likely to survive and continue to grow. Neligan, in his editorial on the future of plastic surgery, outlined concerns about the fate of the specialty. He pointed out that in the United States, interest in reconstructive surgery among plastic surgeons is negligible, and only a few centers are pursuing it. However, he also gave some tips on how to improve the situation. According to the author, a plastic surgeon should be the best at what he does, accessible, collaborative, and innovative. He emphasized the role of teamwork. Complex surgical problems are difficult to solve by a single specialist. Only the cooperation of several can bring the best result. A plastic surgeon is and should be an integral part of the team in fields such as oncology (e.g., breast unit) or traumatology (e.g., orthoplastic procedures). There are still areas where plastic surgery is a pioneer, such as lymphatic surgery. 5

It should be emphasized that plastic surgery is of great importance in global health care and plays a fundamental role, especially in crisis situations, such as mass events, disasters, or wars. This is crucial in complex limb reconstructions, craniofacial injuries, and wound care expertise. 6 7

In summary, there are reasons for concern, especially regarding the migration of specialists to the private sector, which leads to fewer and fewer resources and opportunities for activities in the field of reconstructive surgery. It is necessary to look for a way to retain and motivate young plastic surgery adepts. Adequate motivation and development opportunities appear to be key. 8 Modern plastic surgery, using microsurgical techniques, robotics, drawing on regenerative medicine and tissue engineering, has the potential to evolve, bringing many benefits to patients. For the surgeon, it provides opportunities for unlimited growth and fascination in this wonderful field of medicine.
